# Deciphering NOTCH1 as a Biomarker in Adenoid Cystic Carcinoma: Insights From a Systematic Review With Meta‐Analysis

**DOI:** 10.1111/jop.70028

**Published:** 2025-08-11

**Authors:** Isabela de Sousa Slivar, Bruno de Andrade Zanesco, Manoela Domingues Martins, Leandro Luongo Matos, Fábio Daumas Nunes, Lauren Frenzel Schuch, Vivian Petersen Wagner

**Affiliations:** ^1^ Department of Dentistry School of Dentistry, Universidade de São Paulo (FOUSP) São Paulo Brazil; ^2^ Department of Oral Diagnosis Piracicaba Dental School, Universidade Estadual de Campinas, Piracicaba (UNICAMP) São Paulo Brazil; ^3^ Department of Oral Pathology School of Dentistry, Universidade Federal do Rio Grande do Sul (UFRGS) Porto Alegre Rio Grande do Sul Brazil; ^4^ Departament of Head and Neck Surgery Instituto do Câncer do Estado de São Paulo, Hospital das Clínicas, Faculdade de Medicina da Universidade de São Paulo (ICESP HCFMUSP) São Paulo Brazil; ^5^ Departament of Oral Pathology and Medicine Diagnosis Molecular Pathology Area, School of Dentistry, Universidad de La Republica (UDELAR) Montevideo Uruguay

**Keywords:** biomarkers, head and neck neoplasms, salivary gland neoplasms, tumor

## Abstract

**Background:**

Most studies suggest that NOTCH1 alterations are associated with prognosis in adenoid cystic carcinoma (ACC), but findings remain fragmented. We conducted an integrative analysis to evaluate the prognostic value of NOTCH1‐related biomarkers in ACC.

**Methods:**

A comprehensive search was conducted across multiple databases. Inclusion criteria comprised studies examining NOTCH1‐related features at ACC diagnosis and their relation with survival outcomes. Meta‐analysis was performed on studies sharing similar methodology.

**Results:**

Twelve studies met the eligibility criteria. NOTCH1 mutational status, Notch1 immunoexpression, and NICD1 immunoexpression were associated either with overall, disease‐free or progression‐free survival. Methodological disparities hindered integration of results. Meta‐analyses of HRs were conducted with 2 studies for each index factor, revealing a 2.31‐fold increased risk of death for primary ACC cases with NOTCH1 mutations, a 2.6‐fold increased risk of death for those exhibiting positive NICD1 expression and a 1.91‐fold heightened risk of disease recurrence for cases with high Notch1 expression.

**Conclusion:**

This comprehensive review underscores the prognostic relevance of NOTCH1 in ACC, indicating its potential as a valuable risk stratification tool.

## 1 Introduction

Individuals with rare cancers face numerous challenges, from diagnosis to follow‐up. Low prevalence often limits access to specialists, leading to potential misdiagnoses and delays. Trust in clinical decisions is lower, and evidence‐based therapies and prognostic biomarkers are scarce [1]. Adenoid cystic carcinoma (ACC), with an incidence of 0.35 per 100 000 in the USA, is a slow‐growing yet highly metastatic cancer, often labeled a “slow killer.” Despite modest 5‐year survival rates, ACC requires prolonged follow‐up due to late recurrences, and advanced metastatic cases lack effective systemic treatments [2, 3, 4].

ACC prognosis typically relies on clinical features, such as American Joint Committee on Cancer stage, margin status, tumor architecture, cytology, and perineural invasion [5, 6]. However, ACC lacks robust prognostic biomarkers. In 2017, Ferrarotto et al. [7] found that NOTCH1 mutations define an aggressive ACC subgroup with poor outcomes, proposing nuclear expression of the NOTCH1 intracellular domain (NICD1) as a potential prognostic biomarker. This has been supported by further studies in the United Kingdom and China [8, 9, 10]. In humans, the NOTCH signaling pathway comprises four paralogs (NOTCH1–4) and five canonical ligands: Delta‐like ligands (DLL1, DLL3, and DLL4) and Jagged ligands (JAG1 and JAG2). Ligand binding initiates proteolytic cleavage and release of the NOTCH intracellular domain (NICD), which translocates into the nucleus to form a transcriptional activator complex. NICD can either be degraded in the cytoplasm or transported to the nucleus, where it regulates the transcription of downstream target genes [11]. NOTCH signaling can be activated not only by increased ligand expression but also by structural alterations such as activating mutations. Indeed, previous studies have shown that NICD1 nuclear expression correlates strongly with the presence of NOTCH1 mutations in ACC [7, 12].

Translating biomarkers to clinical use requires rigorous validation. Ideally, prospective trials confirm prognostic value; yet this is challenging with rare cancers [13]. Meta‐analyses provide an alternative by consolidating data from diverse cohorts. Our study aimed to aggregate data linking ACC outcomes with survival.

## 2 Material and Methods

### 2.1 Search Strategy

Electronic searches without publication date restriction were undertaken in October 2023 and then updated in August 2024 in the following databases: PubMed (National Library of Medicine), Web of Science (Thomson Reuters), Scopus (Elsevier), and Embase (Elsevier). The search strategy used was (“Adenoid Cystic Carcinoma” OR “Adenoid Cystic Carcinomas” OR “Carcinomas, Adenoid Cystic” OR “Cystic Carcinoma, Adenoid” OR “Cystic Carcinomas, Adenoid” OR “Adenocystic Carcinoma” OR “Adenocystic Carcinomas” OR “Carcinoma, Adenocystic” OR “Carcinomas, Adenocystic”) AND (NOTCH OR “Receptor, Notch1” OR “Notch1 Protein” OR “Notch1 Receptor” OR NOTCH1) and the exact terms used in each database is detailed in Table S1. Additionally, a grey literature search was performed on Google Scholar and Proquest (reading of the first 100 results). To identify publications that might have been missed, hand searches were further performed by cross‐checking the reference lists of included articles.

### 2.2 Research Question

Our study followed the PICOTS framework, an adaptation of the PICOS strategy for prognostic factor reviews, guided by the CHARMS checklist [14]. P (participants) were ACC patients, I (index factor) was Notch1 investigation, C (comparator) was not assessed, O (outcomes) were recurrence‐free or disease‐specific survival, T (timing) was at diagnosis, and S (setting) was prognostic use at diagnosis.

### 2.3 Screening and Selection

Included articles assessed Notch1's prognostic value (NOTCH1 mutation, Notch1 or NICD1 immunoexpression) in ACC patients for recurrence‐free or disease‐specific survival, without site restrictions. Excluded were conference abstracts, editorials, reviews, case reports, studies with overlapping samples, or those not analyzing Notch1 with survival outcomes individually.

Titles and abstracts identified in electronic searches were reviewed by one author (V.P.W.). Articles meeting eligibility criteria or with unclear abstracts were further assessed via full text. Only studies meeting criteria after full‐text review were included.

Data extraction, guided by a modified CHARMS framework, included participant details (sample size, location, ACC subtype), index test (analysis method), predicted outcome, model development (statistical analysis), and results. Hazard ratios (HRs) with 95% confidence intervals for recurrence‐free and disease‐specific survival, and median survival times for comparison groups were extracted where available.

### 2.4 Critical Appraisal

One author assessed study quality using the Joanna Briggs Institute (JBI) tool [15], evaluating sample quality, criteria, confounders, outcomes, and statistical analysis. The JBI tool was used due to the broader range of study designs included in the review. Responses were categorized as Yes, No, Unclear, or Not/Applicable. Bias risk was rated as High (≤ 49% “yes”), Moderate (50%–69% “yes”), or Low (> 70% “yes”).

### 2.5 Statistical Analysis

All the extracted data were tabulated on Microsoft Office Excel 2019 (Microsoft software) and analyzed descriptively. A meta‐analysis was performed using the R for MacOS software (version 4.3.2, R Foundation for Statistical Computing, Vienna, Austria). Studies heterogeneity was calculated by inconsistency indexes (*I* ^2^), in which *I* ^2^ above 50% was considered an indicator of substantial heterogeneity. As all models achieved an *I* ^2^ of 0% (no significant heterogeneity), a fixed‐effects model was applied.

### 2.6 Other Information

This review was conducted according to the guidelines of the Preferred Reporting Items for Systematic Reviews and Meta‐analyses (PRISMA) Statement [16]. The review was registered at the International Prospective Register of Systematic Reviews (PROSPERO) under the number CRD42023478259.

## 3 Results

### 3.1 Studies Selection

**FIGURE 1:**
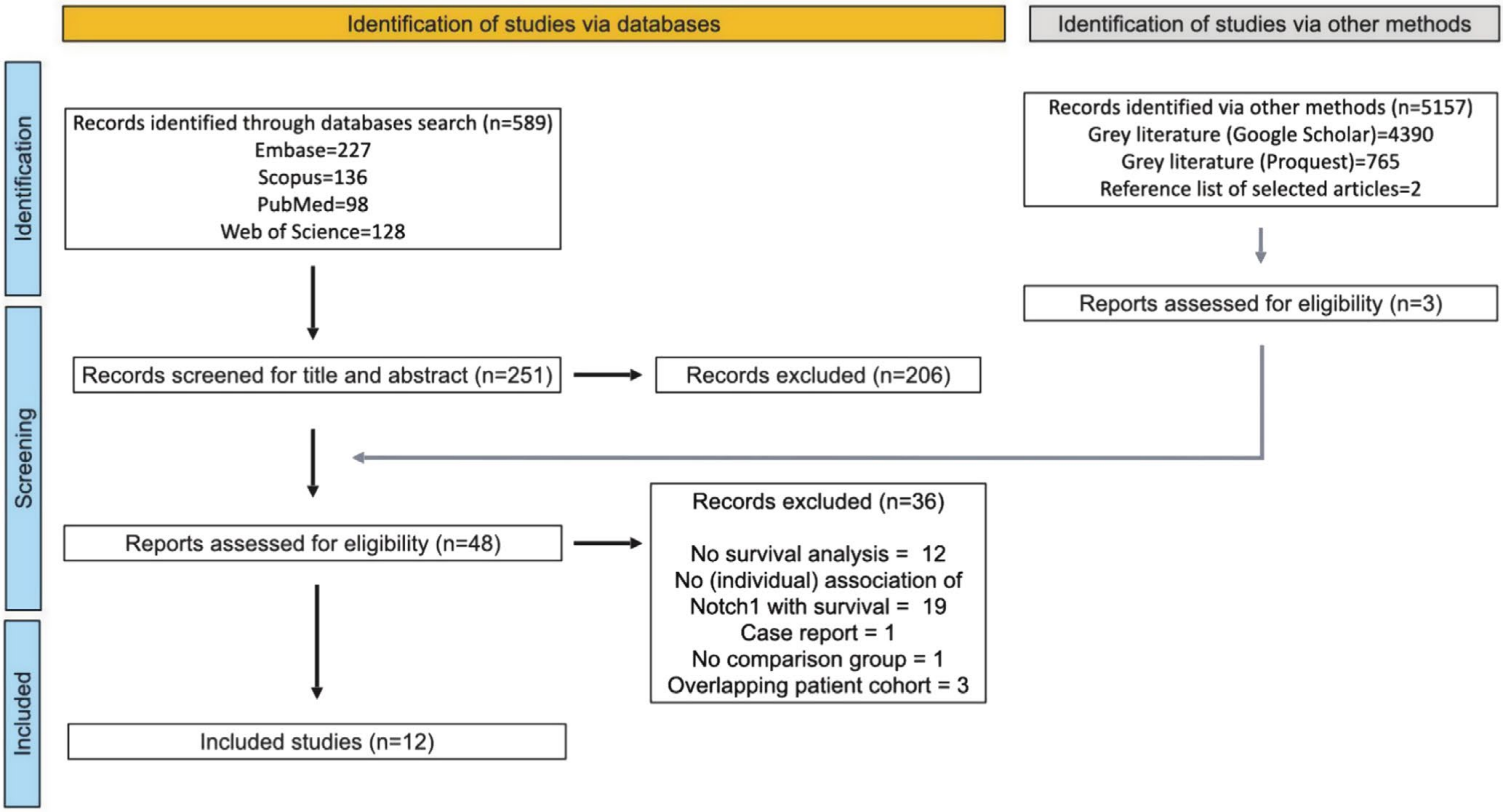
Study flowchart.

The initial electronic search found 589 articles. After removing duplicates (*n* = 310), 222 manuscripts were screened by title and abstract, narrowing to 34 for full‐text review. Two papers were added from references and one from grey literature. After applying inclusion/exclusion criteria, 28 articles were excluded (see Table S2), leaving 12 eligible for data extraction. The flowchart is shown in Figure 1.

### 3.2 Studies General Features

Table 1 summarizes the main characteristics of the studies published from 2017 to 2024, conducted in the USA (*n* = 6), China (*n* = 4), UK (*n* = 1), and India (*n* = 1), with sample sizes from 23 to 368. Most studies focused on salivary gland ACC, with seven exclusively or primarily on these cases. One study each addressed lacrimal gland, breast, and tracheobronchial ACC, whereas two did not specify the site. Additional factors affecting results include tumor status (primary vs. recurrent) and histological grade. Three studies specifically examined Notch1's association with outcomes in recurrent/metastatic cases, and one focused on solid ACC cases.

**TABLE 1:** General features of included studies.

Study	Source of data	Sample size^a^	Location of ACC	Inclusion criteria	Sample type	ACC type			
						Cribriform	Tubular	Solid	Unknown
Anjum et al. [17]	All India Institute of Medical Sciences, New Delhi, India	23	Lacrimal gland	All resected cases	FFPE	14	3	6	—
Feeney et al. [8]	The Christie NHS Foundation Trust, Wilmslow Road, Manchester, UK	120	Salivary glands	Recurrent/metastatic	FFPE	NI	NI	NI	NI
Ferrarotto et al. [7, 18]	Multicenter, USA	120 (WES) 72 (IHQ)	Diverse (most salivary glands)	All resected cases	Fresh‐frozen and FFPE	35	7	36	24
Ferrarotto et al. [19]	Multicenter, USA	54	Diverse (most salivary glands)	All resected cases	Fresh‐frozen and FFPE	21	5	21	7
Ferrarotto et al. [20]	Multicenter, USA	56	Not specified	Recurrent/metastatic	Not specified	NI	NI	NI	NI
Ho et al. [21]	Memorial Sloan Kettering Cancer Center, USA	84	Diverse (most salivary glands)	Recurrent/metastatic	FFPE	NI	NI	NI	NI
Li et al. [22]	The First Affiliated Hospital of Zhengzhou University, Zhengzhou, Henan, China	46	Not specified	Primary	FFPE	NI	NI	NI	NI
Salem et al. [23]	Columbia University Medical, New York, USA	25	Breast ACC	All resected cases	FFPE	4 classic‐type; 13 mixed‐type; 8 solid‐basaloid type			
Sajed et al. 2017	Massachusetts General Hospital and Brigham and Women's Hospital, USA	173 (IHQ)^b^ 29 (NGS)	Diverse (most salivary glands)	All resected cases	FFPE	145	16	16	—
Wang et al. [9]	Ninth People's Hospital affiliated to Shanghai Jiao Tong University, China	49	Head and neck	Most solid cases	FFPE	6		43	—
Xie et al. [10]	Guangzhou Medical University, Tsinghua University, Sun‐Yet Sen University Cancer Center, Sun‐Yet Sen University, and Guangdong General Hospital, China	368	Tracheobronchial	All resected cases	FFPE	294		74	—
Zhang et al. [24]	Peking University School and Hospital of Stomatology, China	125 (IHQ) 16 (PCR)	Salivary glands	All resected cases	Fresh‐frozen and FFPE	62		63	—

Abbreviations: FFPE, formalin‐fixed paraffin‐embedded; IHQ, immunohistochemistry; NGS, next‐generation sequencing; NI, not informed; WES, whole‐exome sequencing.

^a^ Number of cases Notch1 was investigated and further correlated with survival.

^b^ More than one sample per patient was classified for subtype, resulting in sum of ACC subtypes being different from sample size.

### 3.3 Outcomes Investigated and Methods of Investigation

Three Notch1‐related outcomes were associated with survival: NOTCH1 mutation (5 studies), NICD1 immunoexpression (6 studies), and Notch1 immunoexpression (3 studies), as detailed in Tables S2–S4.

NOTCH1 mutation was analyzed via techniques like whole exome sequencing, next‐generation sequencing, and targeted sequencing. Mutation rates ranged from 8.5% in primary cases to 61.2% in solid cases, with R/M cases showing 18%–26% mutation rates.

NICD1 analysis used the D3B8 antibody with varying dilutions. Cut‐offs for positive cases differed: 20%, 70%, and 90% of positive cells, or based on combined cell percentage and staining intensity, with positive cases from 5.6% to 50%.

Notch1 immunoexpression was evaluated using three different antibodies, with positivity defined by staining intensity times the percentage of positive cells. Positive rates were 37.8% and 65%, though one study lacked detailed data.

### 3.4 NOTCH1 Mutation and ACC Prognosis

**FIGURE 2:**
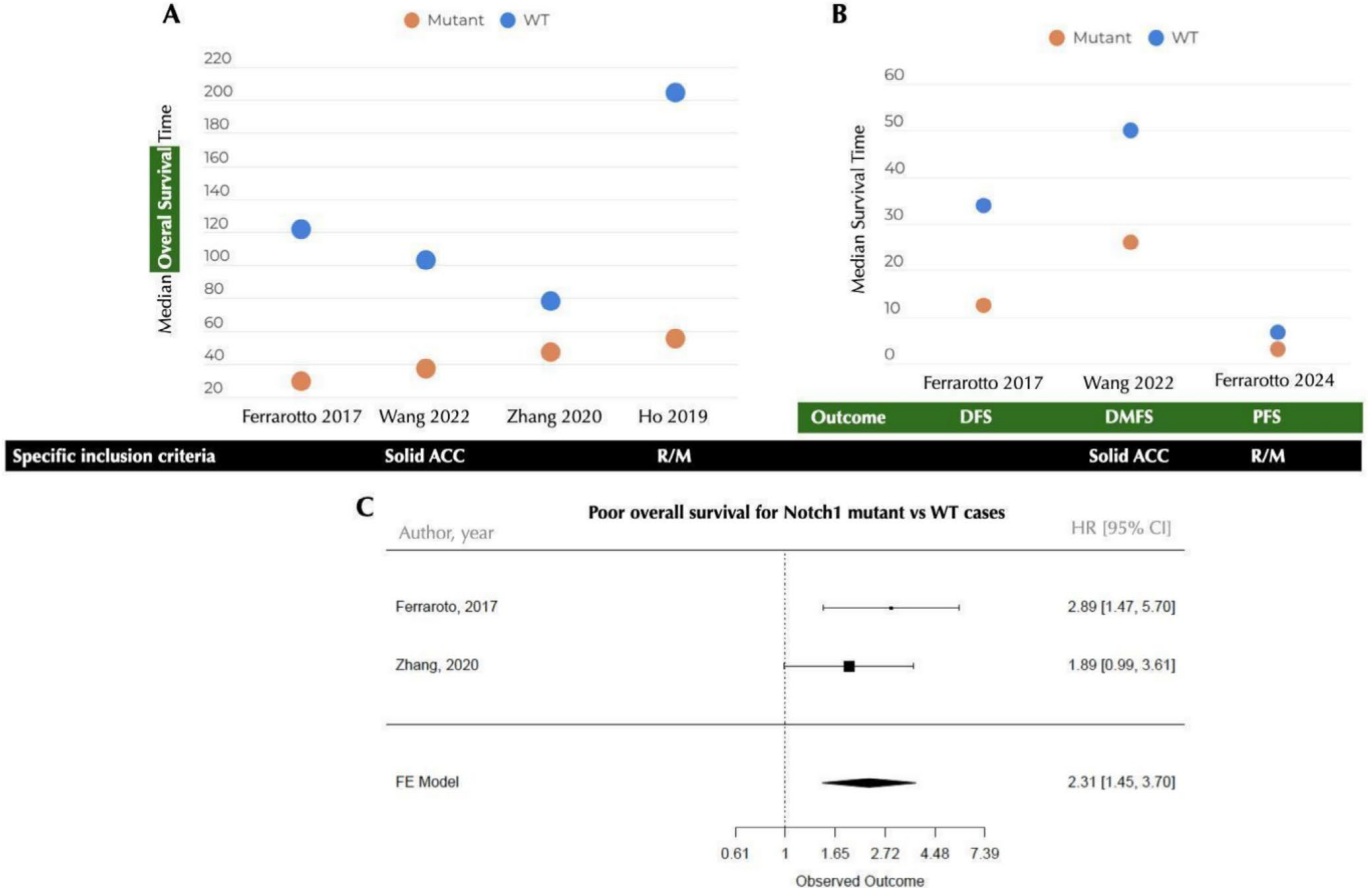
Median survival time of NOTCH1 mutant cases vs. wild type (WT) ACC for (A) overall survival or (B) diverse outcomes (green box). Specific inclusion criteria is highlighted in the black box. (C) Forest plot of NOTCH1 mutation status as a biomarker for overall survival in ACC. CI, confidence interval; DFS, disease free‐survival; DMFS, distant metastasis free survival; HR, hazard ratio; PFS, progression free survival; R/M, recurrent/metastatic.

NOTCH1 mutations were linked to disease‐free, distant metastasis‐free, bone metastasis‐free, progression‐free, and overall survival (Table 2) in samples of 49–125 cases. Analyses used Kaplan–Meier and univariate Cox regression to compare mutated versus wild‐type (WT) cases; one study also analyzed activating versus non‐activating mutations in overall survival. Median survival times with *p* values were shown for groups, with four studies reporting median and three reporting mean survival times. Data varied, and results were compared in Figure 2A,B. HR with 95% confidence intervals were given in three studies; for primary cases, a meta‐analysis (*n* = 2) showed a 2.31‐fold higher death risk in mutated cases (Figure 2C).

**TABLE 2:** Association of NOTCH1 mutational status with survival.

Study	Comparison groups	Outcome	Type of analysis	Median survival time (months)	HR (95% CI)	*p*
Ferrarotto et al. [7, 18]	Notch1 mutation vs. WT	Disease‐free survival	Univariate Cox	12.5 vs. 33.9	2.12 (1.19–3.79)	0.01
	Notch1 mutation vs. WT	Overall survival	Univariate Cox	29.6 vs. 121.9	2.89 (1.47–5.71)	0.002
Ferrarotto et al. [20]	Notch1 mutation vs. WT	Progression‐free survival	Kaplan–Meier	3.1 vs. 6.7	1.67 (0.73–3.85)^a^	—
Ho et al. [21]	Notch1 mutation vs. WT	Overall survival	Kaplan–Meier	55.1 vs. 204.5	—	< 0.001
	Notch1 activating mutation vs. non‐activating	Overall survival	Kaplan–Meier	31.1 vs. 73.8	—	0.04
Wang et al. [9]	Notch1 mutation vs. WT	Overall survival	Kaplan–Meier	37 vs. 103	—	0.001
	Notch1 mutation vs. WT	Distant metastasis‐free survival	Kaplan–Meier	26 vs. 50	—	0.003
	Notch1 mutation vs. WT	Disease‐free survival	Kaplan–Meier	—	—	0.07
Zhang et al. [24]	Notch1 mutation vs. WT	Overall survival	Kaplan–Meier Univariate Cox	47 vs. 78	1.89 (0.99–3.62)	0.04 0.05
	Notch1 mutation vs. WT	Bone metastasis‐free survival	Kaplan–Meier	—	—	0.02

Abbreviations: CI, confidence interval; HR, hazard ratio; WT, wild type.

^a^ The reference group in the study was the mutated cases; so the values here were inverted for comparison purposes.

### 3.5 NICD1 Immunostaining and ACC Prognosis

**FIGURE 3:**
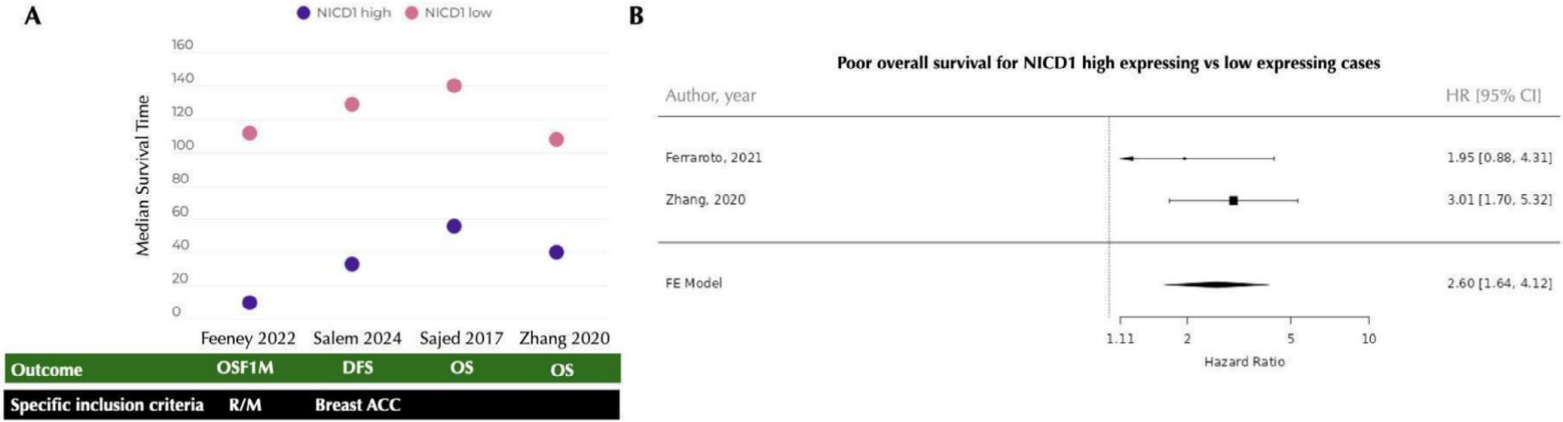
(A) Median survival time of NICD1 high expressing vs. low expressing ACC cases for diverse outcomes (green box). Specific inclusion criteria is highlighted in the black box. (B) Forest plot of NICD1 positivity as a biomarker for overall survival in ACC. CI, confidence interval; DFS, disease free‐survival; HR, hazard ratio; OS, overall survival; OSF1M, overall survival from first metastasis; R/M, recurrent/metastatic.

NICD1 staining was investigated in six studies with samples ranging from 23 to 125 cases (Table S4). Outcomes varied, encompassing overall survival (from the 1st diagnosis or 1st metastasis), bone metastasis‐free survival, and metastasis‐free survival (Table 3). The methods employed for analysis included the Kaplan–Meier method and Cox regressions, both univariate and multivariate. Four studies reported mean overall survival time for positive versus negative cases—three reported based on overall survival from the 1st diagnosis, and one from the 1st metastasis (Figure 3A and Table 3). As inclusion criteria and cut‐off values varied, this data was not combined. The meta‐analysis for the HR was performed based on two studies, as the third study that presented HR data [8] assessed only recurrent/metastatic cases. The final HR based on these two studies indicates a 2.6‐fold increased risk for positive (overexpressing) primary ACC cases to experience death during follow‐up compared to cases with low/negative NICD1 expression (Figure 3B). This analysis was performed with univariate Cox regression results. The HR reported for recurrent/metastatic cases was 6.72 [8].

**TABLE 3:** Association of NICD1 staining with survival.

Study	Outcome	Type of analysis	Median survival time (months)	HR (95% CI)	*p*
Feeney et al. [8]	Overall survival (from diagnosis)	Kaplan–Meier	—	6.72 (0.78–57.53)	< 0.001
	Overall survival (from 1st metastasis)	Kaplan–Meier	9.6 vs. 115.2	15.96 (0.61–413.2)	< 0.001
Ferrarotto et al. [19]	Overall survival	Kaplan–Meier	—	1.95 (0.88–4.3)	0.007
Salem et al. [23]	Disease‐free survival	Kaplan–Meier	33 vs. 129	—	0.04
Sajed et al. [25]	Overall survival	Kaplan–Meier	56.5 vs. 140	—	0.003
Zhang et al. [24]	Overall survival	Kaplan–Meier Univariate Cox Multivariate Cox	40 vs. 108	3.01 (1.70–5.31) 2.87 (1.65–5.10)	< 0.001 < 0.001 0.04
	Bone metastasis‐free survival	Kaplan–Meier	—	—	< 0.001
	Metastasis‐free survival	Kaplan–Meier	—	—	0.01

Abbreviations: CI, confidence interval; HR, hazard ratio.

### 3.6 Notch1 Immunostaining and ACC Prognosis

**FIGURE 4:**
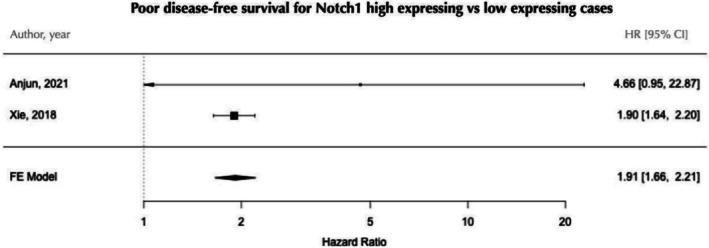
Forest plot of Notch1 positivity as a biomarker for disease‐free survival in ACC. CI, confidence interval; HR, hazard ratio.

The prognostic significance of Notch1 immunohistochemical expression was investigated in three independent studies involving 23, 46, and 368 patients with ACC (Table S5). Two studies employed Cox regression analysis, with one focusing on the association of Notch1 expression with disease‐free survival and the other encompassing both disease‐free and overall survival analyses (Table 4). One study used the Kaplan–Meier method and described a significant association [22]. None of the studies informed the median survival times. Both studies with Coxregression reported HR for disease‐free survival, enabling a meta‐analysis. Pooling the data revealed that patients exhibiting high Notch1 expression faced a 1.91‐fold increased risk of disease recurrence during follow‐up compared to those with negative/low expression levels (Figure 4). This analysis was conducted based on univariate Cox regression results.

**TABLE 4:** Association of Notch1 staining with survival.

Study	Outcome	Type of analysis	Median survival time (months)	HR (95% CI)	*p*
Anjum [17]	Disease‐free survival	Univariate Cox	—	4.66 (0.95–22.88)	0.05
Li [22]	Overall survival	Kaplan–Meier	—	—	0.01
Xie [10]	Disease‐free survival	Univariate Cox	—	1.90 (1.68–2.25)	0.009
	Disease‐free survival	Multivariate Cox	—	1.39 (1.16–1.76)	0.03
	Overall survival	Univariate Cox	—	1.67 (1.38–2.12)	0.01
	Overall survival	Multivariate Cox	—	1.59 (1.27–1.90)	0.01

Abbreviations: CI, confidence interval; HR, hazard ratio.

### 3.7 Reporting Bias

Most studies exhibited a low risk of bias (ranging from 75% to 87.5%). However, one article showed a moderate risk of bias (62.5%), primarily due to partial adherence to criteria in some questions. Comprehensive results of the quality analysis are delineated in Table S6.

## 4 Discussion

The insidious nature of ACC, with a tendency for late distant metastasis, underscores the need for prognostic markers to guide clinical decision‐making and improve patient outcomes. Recent advancements in our understanding of the ACC molecular landscape favor the identification of biomarkers. MYB rearrangements are the most common molecular alterations found in ACC [4]. Yet, despite the high prevalence, its prognostic value appears to be elusive [26]. Mutations in Notch‐pathway‐associated genes are, although less frequent, also recurrent in ACC [27]; studies have indicated an association with a more aggressive phenotype [7, 18]. The current systematic review confirmed that NOTCH1 mutational status and immunohistochemical expression are associated with ACC prognosis within different patient cohorts. Despite the promising findings, the integration of data into meta‐analyses was hampered by studies' diverse methodology, which underscores the need for consistency in reporting prognostic results.

NOTCH1 oncogenic potential was first reported in T cell acute lymphoblastic leukemia in the 90s [28]. Since then, experimental and clinical studies have demonstrated that NOTCH1 is altered in a range of cancer types, including adenocarcinomas originating in the breast, lung, colorectal mucosa, and ovary (reviewed in [29]). Our study focused on clinical data associating NOTCH1 related outcomes with ACC patients' survival. Yet, the clinical evidence surrounding ACC is also further supported by experimental data. As an example, sorted cancer stem cell (CSC) subpopulations of ACC organoids express higher levels of NOTCH1 and NICD1, compared to the unsorted sample [30]. Moreover, NOTCH1 knockdown inhibits spheroidogenesis, confirming its role in ACC CSC maintenance [18]. Another study reported a direct correlation between Notch1 activity and cell proliferation and migration of an ACC cell line [31]. While the organoids have been obtained from ACC validated patient‐derived xenograft (PDX) models, caution must be taken in the analysis of the second study as the cell line used (ACCM) has been shown to be contaminated with HeLa [32]. It is not clear whether the authors confirmed the ACC matching profile of the samples by short tandem repeat (STR) profiling in their study [28].

Much of the data linking NOTCH1 with outcomes in ACC stems from clinical investigations, which either evaluate its prognostic significance or explore tumor responses to NOTCH1 inhibitors. Our objective was to evaluate the biomarker potential of NOTCH1 in ACC through a comprehensive literature review. As a result of our rigid inclusion criteria, only 12 studies were assessed for data extraction. Eleven studies demonstrated a statistically significant association between the investigated factor and ACC prognosis, with variations in outcomes such as disease‐free survival, metastasis‐free survival, or overall survival. One study did not report the statistical results for this specific analysis, as this was not the focus of the study, but the reported mean survival time of mutated cases was smaller in comparison to non‐mutated cases [20]. Overall, the included studies had low risk of bias according to the JBI tool, which enhances the strength of our findings. Although tools such as CHARMS and PROBAST are commonly used in systematic reviews focused specifically on prediction model studies, our review encompassed a broader range of methodological approaches—including experimental, observational, and translational research—where prognostic analyses were often secondary outcomes rather than the primary objective. Given this heterogeneity, the JBI tool offered a flexible and structured framework suitable for appraising studies with varied designs.

Integrating data into meta‐analysis proved challenging due to outcome differences and varied result reporting. Combining outcome probabilities from multiple studies could enhance reliability and reveal cohort‐specific differences. Our three conducted meta‐analyses showed data homogeneity, albeit relying on only two studies each. Validation across diverse cohorts is crucial for consistency. The studies predominantly originated from North America (*n* = 6), Asia (*n* = 5), and Eastern Europe (*n* = 1), highlighting the need for inclusion from other regions like South America, Africa, Western Europe, and Oceania to address potential ethnic influences. Consistent reporting, particularly providing HRs and corresponding 95% confidence intervals from Cox regression, facilitates association strength assessment and data integration. Studies solely reporting median survival time without HRs could not be included in our meta‐analysis.

The prevalence of *NOTCH1* mutated cases ranged from 8.5% [29] to 21.6% [30] among primary ACC cases. We also confirmed from our data that NOTCH1 activating mutations represent the vast majority of events in ACC [7, 9]. Although five studies provided mean survival times for mutant versus WT cases, caution is warranted when interpreting the estimated 3‐fold difference, as significant differences exist in patient characteristics across studies (e.g., primary cases only, solid type only, or recurrent metastatic cases only) and the outcome addressed (overall survival, progression‐free survival [WT]). Our meta‐analysis focused on the two studies with similar inclusion criteria (primary ACC regardless of histological type or stage) and outcomes, both reporting HRs. The analysis revealed that ACC patients with NOTCH1 mutations faced a 2.31‐fold increased risk of death during follow‐up compared to WT cases. We did not evaluate histological subtypes separately in this analysis because the original studies did not report survival outcomes stratified by subtype. However, NOTCH1 signaling has been implicated in determining the differentiation pathways of bipotent epithelial precursors. Aster et al. [11] proposed that MYB‐transformed precursor cells may follow different fates depending on NOTCH1 activation, with gain‐of‐function mutations favoring epithelial over myoepithelial differentiation. Given that ACC exhibits both epithelial and myoepithelial components, this mechanism may influence tumor morphology and behavior. This highlights the importance of future studies integrating molecular and histological data to better understand the prognostic implications of NOTCH1 mutations.

Interestingly, NOTCH1 mutational status was found to be associated not only with overall survival but also with specific outcomes such as disease‐free survival and, notably, PFS in a clinical trial involving R/M disease. The inclusion criteria for this trial required evidence of clinical disease progression within the past 12 months [28]. The trial focused on the effects of PRMT5, a protein known to interact with Notch1 signaling [31]. The average PFS time for the entire cohort was 5.9 months. Patients with NOTCH1 mutations had a shorter PFS of just 3.1 months on average, compared to 6.7 months for non‐mutated cases [28]. In this recently published trial, 30% of patients experienced some reduction in tumor size, and the probability of achieving disease stabilization for more than 6 months was 47% [28]. These results are particularly promising given the current landscape of systemic therapies for ACC [33]. It is becoming increasingly clear that Notch1 could be a valuable therapeutic target, and assessing NOTCH1 status may serve as either a prognostic or predictive biomarker, capable of identifying patients who are more or less likely to respond to specific treatments.

Notch activity is mediated by the release of its intracellular domain (NICD) after Notch ligands activate the membrane‐bound Notch receptor, such as Notch1. NICD translocates to the nucleus where it activates transcription of Notch downstream targets [29]. This review identified that NICD1 was the most common Notch1 associated index factor investigated (*n* = 6). In comparison to Notch1 immunohistochemistry analysis, NICD1 investigation is more valid as it investigates the activated form of the protein. In comparison to NOTCH1 mutational status, immunohistochemistry is more affordable. Moreover, previous studies have demonstrated that NICD1 is significantly correlated with NOTCH1 mutational status in ACC [7, 12]. Yet, NOTCH1 WT tumors can also be NICD1 positive in some instances [7]. This is probably due to deregulation of associated genes or pathways. Whole exome sequencing of ACC cases uncovered that mutations in SPEN, a known regulator of NOTCH signaling, were the second most frequent molecular alteration after MYB‐NFIB [34]. Our review identified a large range in the frequency of NICD1 positivity among primary ACC cases, from 11.2% [25] to 50% [19]. Interestingly, the lowest frequency rate, of only 5.6%, was observed by Feeney et al. [8] in a study comprising only recurrent/metastatic cases [8]. This difference is more likely to be associated with diverse cut‐off points, as most of the data suggest an inverse relationship, in which more aggressive cases overexpress this protein. Our meta‐analysis confirmed this by demonstrating a significant association of NICD1 overexpression and ACC poor overall survival. Notch1 immunostaining was also confirmed to be associated with prognosis, with a significant association in the meta‐analysis with lower disease‐free survival.

The search for biomarkers needs to take into account cost‐effectiveness and reproducibility [13]. Immunohistochemistry carries important advantages regarding cost‐effectiveness. Yet, different from mutation results, which are dichotomous, the staining needs to be analyzed using a consistent method, so the reproducibility aspect is also met. We identified important differences in how NICD1 or Notch1 positivity was determined between studies. Although a significant association with prognosis was observed in all included studies, quantitative determination, by manual cell counting or digital analysis, could help achieve the full potential of this biomarker. Best cut‐off values can be determined based on sensitivity versus specificity, using specific methods such as area under the ROC curve measurements [35]. Once criteria for positivity are clearly determined, it will be important to check reproducibility and confirm the prognostic value within different cohorts.

Although our study contributes valuable insights into the prognostic potential of NOTCH1 in ACC, some limitations must be acknowledged. The scarcity of studies meeting our inclusion criteria constrained the scope of our meta‐analysis, highlighting the need for more comprehensive investigations in this area. Furthermore, the final HRs of the meta‐analysis were influenced by studies with narrower confidence intervals, potentially skewing the overall estimations. It is essential to emphasize that the results presented herein are not definitive. The limited number of studies available for analysis and the heterogeneity in methodologies and outcome reporting hampered a more definite response.

## 5 Conclusions

Our findings confirm a significant link between NOTCH1 mutations, immunohistochemical expression, and survival outcomes in ACC. However, integrating data into meta‐analyses was challenging due to methodological variations, highlighting the need for standardized reporting in prognostic studies. Despite these issues, the consistent association across patient cohorts supports NOTCH1's potential as a prognostic marker in ACC.

## Author Contributions

**Isabela de Sousa Slivar:** data acquisition, data analysis and interpretation, manuscript preparation, manuscript review. **Bruno de Andrade Zanesco:** data acquisition, data analysis and interpretation, manuscript preparation, manuscript review. **Manoela Domingues Martins:** study concepts, manuscript editing, manuscript review. **Leandro Luongo Matos:** study concepts, manuscript editing, manuscript review. **Fábio Daumas Nunes:** study concepts, manuscript editing, manuscript review. **Lauren Frenzel Schuch:** study design, quality control of data and algorithms, data analysis and interpretation, manuscript editing, manuscript review. **Vivian Petersen Wagner:** study concepts, study design, statistical analysis, manuscript preparation, manuscript review.

## Conflicts of Interest

The authors declare no conflicts of interest.

## Peer Review

The peer review history for this article is available at https://www.webofscience.com/api/gateway/wos/peer‐review/10.1111/jop.70028.

## Supporting information


**Table S1:** Search strategies.
**Table S2:** Full‐text results analysis with reasons for exclusion.
**Table S3:** Methods and overall results of studies investigating Notch1 mutation as a biomarker in ACC.
**Table S4:** Methods and overall results of studies investigating NICD1 expression by IHQ as a biomarker in ACC.
**Table S5:** Methods and overall results of studies investigating Notch1 expression by IHQ as a biomarker in ACC.
**Table S6:** Report of bias analysis.

## Data Availability

The data that support the findings of this study are available from the corresponding author upon reasonable request.
