# Supplementary material for: Deciphering NOTCH1 as a Biomarker in Adenoid Cystic Carcinoma: Insights From a Systematic Review With Meta‐Analysis
Source: J Oral Pathol Med. 2025 Aug 11;54(8):647–57. doi: 10.1111/jop.70028 (PMC12419985; doi:10.1111/jop.70028)
Supplement: Supplementary file 1 — Table S1: Search strategies. Table S2: Full‐text results analysis with reasons for exclusion. Table S3: Methods and overall results of studies investigating Notch1 mutation as a biomarker in ACC. Table S4: Methods and overall results of studies investigating NICD1 expression by IHQ as a biomarker in ACC. Table S5: Methods and overall results of studies investigating Notch1 expression by IHQ as a biomarker in ACC. Table S6: Report of bias analysis. [file JOP-54-647-s001.docx]

**Supplementary Tables**

**Supplementary Table 1**. Search strategies

| Database | Search strategy |
| --- | --- |
| Pubmed | ("Adenoid Cystic Carcinoma" OR "Adenoid Cystic Carcinomas" OR "Carcinomas, Adenoid Cystic" OR "Cystic Carcinoma, Adenoid" OR "Cystic Carcinomas, Adenoid" OR "Adenocystic Carcinoma" OR "Adenocystic Carcinomas" OR "Carcinoma, Adenocystic" OR "Carcinomas, Adenocystic") AND (NOTCH OR "Receptor, Notch1" OR "Notch1 Protein" OR "Notch1 Receptor" OR NOTCH1) |
| Scopus | (TITLE-ABS-KEY (notch OR "Receptor, Notch1" OR "Notch1 Protein" OR "Notch1 Receptor" OR notch1 ) AND TITLE-ABS-KEY ( "Adenoid Cystic Carcinoma" OR "Adenoid Cystic Carcinomas" OR "Carcinomas, Adenoid Cystic" OR "Cystic Carcinoma, Adenoid" OR "Cystic Carcinomas, Adenoid" OR "Adenocystic Carcinoma" OR "Adenocystic Carcinomas" OR "Carcinoma, Adenocystic" OR "Carcinomas, Adenocystic" ) ) |
| Web of science | “Adenoid Cystic Carcinoma” OR “Adenoid Cystic Carcinomas” OR “Carcinomas, Adenoid Cystic” OR “Cystic Carcinoma, Adenoid” OR “Cystic Carcinomas, Adenoid” OR “Adenocystic Carcinoma” OR “Adenocystic Carcinomas” OR “Carcinoma, Adenocystic” OR “Carcinomas, Adenocystic” (All Fields) and NOTCH OR “Receptor, Notch1” OR “Notch1 Protein” OR “Notch1 Receptor” OR NOTCH1 (All Fields) |
| Embase | (notch OR 'receptor, notch1' OR 'notch1 protein' OR 'notch1 receptor' OR notch1) AND ('adenoid cystic carcinoma' OR 'adenoid cystic carcinomas' OR 'carcinomas, adenoid cystic' OR 'cystic carcinoma, adenoid' OR 'cystic carcinomas, adenoid' OR 'adenocystic carcinoma' OR 'adenocystic carcinomas' OR 'carcinoma, adenocystic' OR 'carcinomas, adenocystic') |
| Grey literature (ProQuest and Google Scholar) | (NOTCH OR “Receptor, Notch1” OR “Notch1 Protein” OR “Notch1 Receptor” OR NOTCH1) AND (“Adenoid Cystic Carcinoma” OR “Adenoid Cystic Carcinomas” OR “Adenoid Cystic Carcinoma” OR “Adenoid Cystic Carcinomas”) |

**Supplementary Table 2**. Full-text reading and eligibility criteria application



| First Author | Year | Title | Journal | Included | Reason for exclusion |
| --- | --- | --- | --- | --- | --- |
| Alerraqi, E | 2023 | Sinonasal Hyalinizing Adenoid Cystic Carcinoma Is Molecularly Different from Its Salivary and Breast Counterparts | JOURNAL OF MOLECULAR PATHOLOGY | No | No association of NOTCH1 with survival / outcomes |
| Anjum, S. | 2021 | Prognostic impact of Notch1 receptor and clinicopathological High-Risk Predictors in lacrimal gland adenoid cystic carcinoma | Acta Ophthalmol | Yes | - |
| Bell, D. | 2014 | Expression and significance of notch signaling pathway in salivary adenoid cystic carcinoma | Ann Diagn Pathol | No | No description of NOTCH1 association with survival |
| Brayer, K. J. | 2023 | Dominant Gene Expression Profiles Define Adenoid Cystic Carcinoma (ACC) from Different Tissues: Validation of a Gene Signature Classifier for Poor Survival in Salivary Gland ACC | Cancers | No | No association of NOTCH1 with survival / outcomes |
| de Mendonça, R. P. | 2020 | Role of hypoxia-related proteins in adenoid cystic carcinoma invasion | Diagn Pathol | No | No association of NOTCH1 with survival / outcomes |
| Feeney, L. | 2022 | Clinical disease course and survival outcomes following disease recurrence in adenoid cystic carcinoma with and without NOTCH signaling pathway activation | Oral Oncol | Yes | - |
| Ferrarotto, R | 2024 | PRT543, a protein arginine methyltransferase 5 inhibitor, in patients with advanced adenoid cystic carcinoma: An open-label, phase I dose-expansion study | ORAL ONCOLOGY | Yes | - |
| Ferrarotto, R. | 2017 | Activating NOTCH1 Mutations Define a Distinct Subgroup of Patients With Adenoid Cystic Carcinoma Who Have Poor Prognosis, Propensity to Bone and Liver Metastasis, and Potential Responsiveness to Notch1 Inhibitors | J Clin Oncol | Yes | - |
| Ferrarotto, R. | 2021 | Proteogenomic Analysis of Salivary Adenoid Cystic Carcinomas Defines Molecular Subtypes and Identifies Therapeutic Targets | Clin Cancer Res | Yes | - |
| Frerich, C. A. | 2018 | Transcriptomes define distinct subgroups of salivary gland adenoid cystic carcinoma with different driver mutations and outcomes | Oncotarget | No | No association of NOTCH1 with survival / outcomes |
| Frierson Jr, H. F. | 2013 | Mutation signature of adenoid cystic carcinoma: Evidence for transcriptional and epigenetic reprogramming | Journal of Clinical Investigation | No | No association of NOTCH1 with survival / outcomes |
| Haider, S. M. | 2024 | Clinical outcomes of NOTCH pathway-activated adenoid cystic carcinoma with and without co-occurrent TP53 mutation | Journal of Clinical Oncology | No | No analysis of NOTCH1 mutated cases isolated from other mutated cases |
| Hanna, G. J. | 2020 | Long-term outcomes and clinicogenomic correlates in recurrent, metastatic adenoid cystic carcinoma | Oral Oncol | No | No analysis of NOTCH1 mutated cases isolated from other mutated cases |
| Hanna, G. J. | 2024 | Molecular Profiling and the Impact of Treatment on Outcomes in Adenoid Cystic Carcinoma Type I and II | Clin Cancer Res | No | No association of NOTCH1 with survival / outcomes |
| Hanna, GJ | 2023 | A Phase I Study of the Pan-Notch Inhibitor CB-103 for Patients with Advanced Adenoid Cystic Carcinoma and Other Tumors | CANCER RESEARCH COMMUNICATIONS | No | No association of NOTCH1 with survival / outcomes |
| Ho, A. S. | 2013 | The mutational landscape of adenoid cystic carcinoma | Nature Genetics | No | No association of NOTCH1 with survival / outcomes |
| Ho, A. S. | 2019 | Genetic hallmarks of recurrent/metastatic adenoid cystic carcinoma | J Clin Invest | Yes | - |
| Huang, J. | 2024 | MYB alternative promoter activity is increased in adenoid cystic carcinoma metastases and is associated with a specific gene expression signature | Oral Oncol | No | No association of NOTCH1 with survival / outcomes |
| Iwaki, S. | 2024 | Comprehensive genomic profiling of salivary gland carcinoma: Analysis of the Center for Cancer Genomics and Advanced Therapeutics database in Japan | International Journal of Cancer | No | No association of NOTCH1 with survival / outcomes |
| Karpinets, T. V. | 2021 | Whole-Genome Sequencing of Common Salivary Gland Carcinomas: Subtype-Restricted and Shared Genetic Alterations | Clin Cancer Res | No | No association of NOTCH1 with survival / outcomes in ACC |
| Kieran, R. | 2021 | Response of NOTCH1-Activated Tracheal Adenoid Cystic Carcinoma to the Gamma Secretase Inhibitor Nirogacestat | JCO Precis Oncol | No | Case report |
| Lassche, G. | 2022 | Identification of Fusion Genes and Targets for Genetically Matched Therapies in a Large Cohort of Salivary Gland Cancer Patients | Cancers (Basel) | No | No survival analysis |
| Li, M. | 2018 | Mutational landscape and clonal diversity of pulmonary adenoid cystic carcinoma | Cancer Biol Ther | No | No survival analysis |
| Li, R | 2024 | Anti-NOTCH1 therapy with OMP-52 M51 inhibits salivary adenoid cystic carcinoma by depressing epithelial-mesenchymal transition (EMT) process and inducing ferroptosis | OXICOLOGY AND APPLIED PHARMACOLOGY | Yes | - |
| Martelotto, L. G. | 2015 | Genomic landscape of adenoid cystic carcinoma of the breast | J Pathol | No | No survival analysis |
| Massé, J. | 2020 | Solid-type adenoid cystic carcinoma of the breast, a distinct molecular entity enriched in NOTCH and CREBBP mutations | Mod Pathol | No | No survival analysis |
| Morris, L. G. T. | 2017 | The Molecular Landscape of Recurrent and Metastatic Head and Neck Cancers: Insights From a Precision Oncology Sequencing Platform | JAMA Oncol | No | No survival analysis |
| Parikh, A. S. | 2022 | Report Single-cell RNA sequencing identifies a paracrine interaction that may drive oncogenic notch signaling in human adenoid cystic carcinoma | Cell Reports | No | No survival analysis |
| Patel, K. | 2023 | Gene expression patterns in adenoid cystic carcinoma with and without diffuse NOTCH1 intracellular domain (NICD1) immunohistochemistry staining | Oral Oncol | No | NCD1 staining in same cohort from Feeney 2022 |
| Rettig, E. M. | 2016 | Whole-Genome Sequencing of Salivary Gland Adenoid Cystic Carcinoma | Cancer Prev Res (Phila) | No | No survival analysis |
| Ross, J. S. | 2014 | Comprehensive genomic profiling of relapsed and metastatic adenoid cystic carcinomas by next-generation sequencing reveals potential new routes to targeted therapies | Am J Surg Pathol | No | No survival analysis |
| Sajed, D. P. | 2017 | Diffuse Staining for Activated NOTCH1 Correlates With <i>NOTCH1</i> Mutation Status and Is Associated With Worse Outcome in Adenoid Cystic Carcinoma | American Journal of Surgical Pathology | Yes | - |
| Salem, A | 2024 | Impact of NOTCH1 expression in primary breast adenoid cystic carcinoma | JOURNAL OF CLINICAL PATHOLOGY | Yes | - |
| Sant, D. W. | 2017 | Whole Exome Sequencing of Lacrimal Gland Adenoid Cystic Carcinoma | Invest Ophthalmol Vis Sci | No | No survival analysis |
| Schwartz, C. J. | 2022 | The clinical behavior and genomic features of the so-called adenoid cystic carcinomas of the solid variant with basaloid features | Mod Pathol | No | No association of NOTCH1 with survival / outcomes |
| Shamir, E. R. | 2023 | Solid-Basaloid Adenoid Cystic Carcinoma of the Breast: An Aggressive Subtype Enriched for Notch Pathway and Chromatin Modifier Mutations With MYB Overexpression | Mod Pathol | No | No association of NOTCH1 with survival / outcomes |
| Shamir, E. R. | 2023 | Solid-Basaloid Adenoid Cystic Carcinoma of the Breast: An Aggressive Subtype Enriched for Notch Pathway and Chromatin Modifier Mutations With MYB Overexpression | Modern Pathology | No | No association of NOTCH1 with survival / outcomes |
| Stoeck, A. | 2014 | Discovery of biomarkers predictive of GSI response in triple-negative breast cancer and adenoid cystic carcinoma | Cancer Discov | No | No association of NOTCH1 with survival / outcomes |
| Su, B. H. | 2014 | NOTCH1 signaling contributes to cell growth, anti-apoptosis and metastasis in salivary adenoid cystic carcinoma | Oncotarget | No | NOTCH1 IHQ staining not correlated with survival |
| Thierauf, J. | 2019 | Clinically Integrated Molecular Diagnostics in Adenoid Cystic Carcinoma | Oncologist | No | No analysis of NOTCH1 mutated cases isolated from other mutated cases |
| Wang, F. | 2021 | Mutational landscape of primary pulmonary salivary gland-type tumors through targeted next-generation sequencing | Lung Cancer | No | No survival analysis |
| Wang, K. | 2021 | Dll4/Notch1 signalling pathway is required in collective invasion of salivary adenoid cystic carcinoma | Oncol Rep | No | No survival analysis |
| Wang, Y. | 2022 | Genetic heterogeneity and therapeutic target detection through microdissection in solid-type adenoid cystic carcinoma | Pathology | Yes | - |
| Xie, M. | 2018 | The Prognostic Significance of Notch1 and Fatty Acid Binding Protein 7 (FABP7) Expression in Resected Tracheobronchial Adenoid Cystic Carcinoma: A Multicenter Retrospective Study | Cancer Res Treat | Yes | - |
| Zhang, Y. | 2020 | Notch activation leads to loss of myoepithelial differentiation and poor outcome in solid adenoid cystic carcinoma | Oral Dis | Yes | - |
| Zhang, Y. | 2023 | Notch activation promotes bone metastasis via SPARC inhibition in adenoid cystic carcinoma | Oral Dis | No | Overlap of more than 50% of the sample from previous study |
| Zhang, Y. | 2024 | Notch activation promotes bone metastasis via SPARC inhibition in adenoid cystic carcinoma | Oral Diseases | No | Overlap of more than 50% of the samble from previous study |
| Zhao, Z. L. | 2015 | Notch signaling induces epithelial-mesenchymal transition to promote invasion and metastasis in adenoid cystic carcinoma | Am J Transl Res | No | No survival analysis |

**Supplementary Table 3**. Methods and overall results of studies investigating Notch1 mutation as a biomarker in ACC

| **Author(s), Year** | **Number of cases** | **Method** | **Overall results** |
| --- | --- | --- | --- |
| Ferrarotto et al., 2017 | 102 | WES (n=70)  Targeted sequencing for gene panel that included NOTCH1 (n=32) | Frequency of 14.70% mutant cases (13.70% activating mutations). Mutant cases were more likely to have solid histology, present with advanced-stage disease, developing metastasis in the liver or bone. Relapse-free and OS were shorter in mutant cases. |
| Ferrarotto et al., 2024 | 56 R/M cases | Not specified | Frequency of 22.5% mutant cases amongst R/M cases with available information regarding NOTCH mutation status (n=49). Mutant cases had shorter progression free survival time in this clinical trial. |
| Ho et al., 2019 | 177 primary and 868 R/M cases (84 with follow-up) | NGS | Frequency of 8.5% and 26.3% amongst primary and R/M cases respectively. In R/M cases, 18.3% of cases had activating mutations. Notch1 R/M mutant cases had poor OS and among mutant cases, activating mutations were also associated with poor OS. |
| Wang et al., 2022 | 49 (solid cases) | NGS | Frequency of 61.2% mutated cases (55.1% activating mutations). Mutant cases had lower OS and distant metastasis free survival rates. No differences in recurrence-free survival. |
| Zhang et al., 2020 | 125 | DNA sequencing | Frequency of 21.6% of cases with activating mutations. Mutant cases were more likely to be of solid subtype, had advanced clinical stage, and presented poor OS and bone metastasis free survival. |

R/M – recurrent metastatic; WES – whole exome sequencing; NGS – next generation sequencing; OS – overall survival

**Supplementary Table 4**. Methods and overall results of studies investigating NICD1 expression by IHQ as a biomarker in ACC

| **Author(s), Year** | **Number of cases** | **Clone, dilution** | **Criteria** | **Overall results** |
| --- | --- | --- | --- | --- |
| Anjun et al., 2021 | 23 | D3B8, 1:50 | Subset positivity (<90% cells) or diffusely positive (>90% cells) | Frequency of 39% of positive cases. No association with clinicopathologic parameters or survival. |
| Feeney et al., 2022 | 88 | D3B8, NI | Positive = diffusely positive nuclear staining | Frequency of 5.6% of positive cases. Significant association with poor OS from diagnosis and from first recurrence. |
| Ferrarotto et al., 2021 | 50 | D3B8, NI | Positive (nuclear staining in ≥70% of tumor cells) or negative (nuclear staining in <70% tumor cells) | Overall frequency of 50% of positive cases. Positive cases associated with MYC-p63 profile and had poor OS. |
| Salem et al., 2024 | 24 | D3B8, NI | Negative score = 0 (<1%), or 1+ (<20%); positive score = 2+ (20%–50%), or 3+ (>50%) based only on nuclear staining. | Overall frequency of 44% of positive cases. Positive cases associated with larger size, recurrence or metastasis and poor disease free survival. |
| Sajed et al., 2017 | 177 (27 with follow-up data) | D3B8, 1:50 | Subset positivity (<90% cells) or diffusely positive (>90% cells) | Frequency of 11.2% diffusely positive cases, which were more likely to be of solid subtype and present MYB rearrangements. Significant association with poor OS. |
| Zhang et al., 2020 | 125 cases | D3B8, 1:100 | Intensity (0, negative; +1, weak; +2, moderate; +3, strong) multiplied by percentage of positive cells (1%; 1, 2%–25%; 2, 26%–50%; 3, 51%–75%). Final score above 5 was considered a high expression. | Frequency of 38.4% cases with high expression. Significant association with Notch1 activating mutations, solid growth pattern and loss of myoepithelial differentiation marked by p63. Association with poor metastasis-free survival, bone-metastasis free survival and OS. |

OS – overall survival; NI – not informed

**Supplementary Table 5**. Methods and overall results of studies investigating Notch1 expression by IHQ as a biomarker in ACC

| **Author, Year** | **Number of cases** | **Clone, dilution** | **Criteria** | **Overall results** |
| --- | --- | --- | --- | --- |
| Anjun et al., 2021 | 23 | D1E11  XP, 1:200 | Staining (0, no staining; 1+, weak staining; 2+, moderate; 3+, strong) multiplied by percentage positivity (0 - no positive cells; 1 ˂10%; 2 - 10 –50%; and 3 ˃50%). Final score of ≥4 was defined as positive/overexpression. | Frequency of 65% of positive cases. Significant association with disease free survival. No association on multivariate analysis. |
| Li et al., 2024 | 46 | EP1238Y; NI | Not specified | ACC samples exhibited much higher NOTCH1 expression levels than that of the paired normal specimens. ACC patients with high levels of NOTCH1 were showed  poorer OS. |
| Xie et al., 2018 | 368 | Val1744, NI | Intensity (0, negative; +1, weak; +2, moderate; +3, strong) multiplied by percentage of positive cells (< 25%, 25%-50%, and > 50%). Final score > 150 = high expression | Frequency of 37.8% of cases with high expression, which was associated with lymph node involvement, bigger tumor size, presence of MYB-NFIB fusion, poor recurrence-free survival, and OS. |

OS – overall survival; NI – not informed

| Author (s), year of publication | Were the criteria for inclusion in the sample clearly defined? | Were the study subjects and the setting described in detail? | Was the exposure measured in a valid and reliable way? | Were objective, standard criteria used for measurement of the condition? | Were confounding factors identified? | Were strategies to deal with confounding factors stated? | Were the outcomes measured in a valid and reliable way? | Was appropriate statistical analysis used? | Risk of bias (%) |
| --- | --- | --- | --- | --- | --- | --- | --- | --- | --- |
| Ferrarotto et al., 2017 | Yes | Yes | Yes | Yes | Yes | No | Yes | Yes | **Low**  **(87.5)** |
| Sajed et al., 2017 | Yes | Yes | Yes | Yes | Yes | No | Yes | Yes | **Low**  **(87.5)** |
| Xie et al., 2018 | Yes | Partial | Yes | Yes | Yes | No | Yes | Yes | **Low**  **(75)** |
| Ho et al., 2019 | Yes | Partial | Yes | Yes | Yes | Yes | Yes | Yes | **Low**  **(87.5)** |
| Zhang et al., 2020 | Yes | Partial | Yes | Yes | Yes | Yes | Yes | Yes | **Low**  **(87.5)** |
| Anjum et al., 2021 | Yes | Yes | Yes | Yes | Yes | No | Yes | Yes | **Low**  **(87.5)** |
| Ferrarotto et al., 2021 | Yes | Yes | Yes | Yes | Yes | No | Yes | Yes | **Low**  **(87.5)** |
| Fenney et al., 2022 | Yes | Partial | Yes | Yes | Yes | No | Yes | Yes | **Low**  **(75)** |
| Wang et al., 2022 | Yes | Partial | Yes | Yes | Yes | No | Yes | Yes | **Low**  **(75)** |
| Ferrarotto et al., 2024 | Yes | Partial | Yes | Yes | Yes | No | Yes | Yes | **Low (75)** |
| Salem et al., 2024 | Yes | Yes | Yes | Yes | Yes | No | Yes | Yes | **Low (87.5)** |
| Li et al., 2024 | Yes | Partial | Yes | Yes | Yes | No | Partial | Yes | **Moderate (62.5)** |

**Supplementary Table 6**. Critical appraisal of the included articles
